# Attitudes towards prescribing cognitive enhancers among primary care physicians in Germany

**DOI:** 10.1186/1471-2296-15-3

**Published:** 2014-01-08

**Authors:** Andreas G Franke, Carolin Papenburg, Elena Schotten, Peter B Reiner, Klaus Lieb

**Affiliations:** 1 Department of Psychiatry and Psychotherapy, University Medical Centre Mainz, Untere Zahlbacher Str. 17, D – 55128 Mainz, Germany; 2 University of Neubrandenburg, University of Applied Sciences, Brodaer Str. 2, D – 17033, Neubrandenburg, Germany; 3 National Core for Neuroethics, University of British Columbia, 2211 Wesbrook Mall, Koerner Pavilion, Room S 124, Vancouver, British Columbia, Canada

**Keywords:** Cognitive enhancement, Primary care physician(s), Prescription drug(s)

## Abstract

**Background:**

Primary care physicians are gate keepers to the medical system having a key role in giving information and prescribing drugs to their patients. In this respect they are involved in claims of patients/clients for pharmacological Cognitive Enhancement (CE). Therefore, we studied the knowledge of primary care physicians about CE and their attitudes toward prescribing CE drugs to healthy subjects.

**Methods:**

A self-report paper-and-pencil questionnaire and case vignettes describing a hypothetical CE drug were sent out to all 2,753 registered primary care physicians in Rhineland Palatine, Germany. 832, i.e. 30.2% filled in the questionnaire anonymously.

**Results:**

96.0% of all participating physicians had already heard about CE. However, only 5.3% stated to be very familiar with this subject and 43.5% judged themselves as being not familiar with CE. 7.0% had been asked by their clients to prescribe a drug for CE during the last week, 19.0% during the last month, and 40.8% during the last year. The comfort level to prescribe CE drugs was very low and significantly lower than to prescribe sildenafil (Viagra®). Comfort level was mainly affected by the age of the client asking for prescription of CE drugs, followed by the availability of non-pharmacological alternatives, fear of misuse of the prescribed drug by the client and the missing indication of prescribing a drug.

**Conclusions:**

Although a relatively high proportion of primary care physicians have been asked by their clients to prescribe CE drugs, only a small proportion are well informed about the possibilities of CE. Since physicians are gate keepers to the medical system and have a key role regarding a drugs’ prescription, objective information should be made available to physicians about biological, ethical and social consequences of CE use.

## Background

Primary care physicians play a crucial role in prescribing drugs. In many jurisdictions they are the first individuals who have to be contacted by the general public when a prescription is sought. Regarding covering costs by the health care system, in the United Kingdom patients have to register at a primary care physician who gives referrals to specialists. In Germany, patients can chose and change their primary care physician who gives referrals; however, patients have the opportunity to contact a specialist directly. In the United States, patients are able to obtain an appointment with a specialist without a referral from a primary care physician.

As such, depending on the national health care system, primary care physicians act more or less as “gatekeepers” for the medical system. Even if prescription drugs against somatic disorders are the most prevalently prescribed drugs among primary care physicians (e.g. non-steroidal anti-inflammatories, beta-blockers, etc.), they frequently are contacted for advice and counselling for mental disorders [1]. With particular reference to the phenomenon of pharmacological cognitive enhancement (CE), Hotze and colleagues found that two thirds of surveyed primary care physicians received requests for “medicine or services that the physicians considered to be for enhancement rather than therapy” monthly and 12.0% at least daily. This survey among a random sample of 1,500 practicing physicians from the American Medical Association (AMA) Masterfile found that 37.0% of the participants stated that they prescribed what they viewed as medicine for enhancement at least monthly and 4.0% at least daily [2].

The group of substances used by healthy subjects for CE purposes consists of over-the-counter- (OTC-) drugs (e.g. Ginkgo biloba, caffeine tablets, etc.) as well as illicit and prescription drugs. Among the drugs that require a prescription are stimulants such as methylphenidate (MPH) and amphetamines (AMPH) such as Adderall®, as well as other drugs such as modafinil, anti-dementia drugs, beta-blockers, and antidepressants for mood enhancement (ME) [3-6]. In Germany, the prescription volume of MPH, indicated for attention deficit hyperactivity disorder (ADHD), has risen from 20 million daily doses in 2003 to 58 million daily doses in 2012 which is hardly to explain with the increase of diagnoses of ADHD; the prescription volume of Atomoxetin, however, is stable for the last years [7].

Bergström and Lynöe found that more than 80.0% of 300 surveyed primary care physicians in Sweden were convinced that CE drugs should not be covered by the health insurance system and individuals should pay themselves for CE drugs [8]. In a web-based survey of 212 primary care physicians throughout the United States and Canada, Banjo and colleagues found that nearly 2/3 of the participants had already read articles about CE in the public or scientific media and yet just over half rated themselves as not being familiar with the topic of CE [9].

Previous studies on CE found large differences in prevalence rates for CE use ranging from > 1 – 20% among students [10-19]. Using the Randomized Response Technique (RRT) which guarantees an especially high degree of privacy, anonymity, and confidentiality, we found in German students a one-year prevalence rate of 20.0% for the use of “pharmaceuticals or illegal drugs which you cannot buy in a drug-store and which were not prescribed to you to treat a disease”. [11]. An anonymous online survey by the journal “Nature” depicted a lifetime prevalence rate of 20.0% for prescription drugs such as beta-blockers, modafinil and methylphenidate (MPH), stressing that MPH is the most popular substance used for CE [20]. Another study by our group using RRT among surgeons revealed a lifetime prevalence rate of 19.9% for the use of prescription and illicit drugs for CE purposes and a lifetime prevalence rate of 15.1% for the use of prescription antidepressants for ME [21].

The present study presents data on the knowledge of, attitudes towards, familiarity with, frequency of being asked for, and comfort levels with prescribing CE drugs by primary care physicians in Germany.

## Methods

### Participants and procedure

Envelopes with paper-and-pencil questionnaires were sent out in June 2011 to all registered primary care physicians (n = 2,753) in Rhineland Palatine, a state in West Germany with about four million inhabitants. Participants were asked to complete the questionnaire anonymously and to return it in an anonymous, addressed and pre-paid envelope within one month. Physicians who did not answer were identified using a code, called twice via telephone and asked to send back the questionnaire. Those who did not possess the questionnaire any more were sent the questionnaire again. Participants also had the option of returning the questionnaires via email or fax.

The first wave of completed questionnaires were received between July and September 2011. Afterwards, the questionnaires were returned according to the order of the telephone calls.

530 physicians responded immediately by sending back their questionnaire (first response rate: 19.3%), 302 responded after telephone calls (second response rate: 11.0%) resulting in a total response rate of 832 (30.2%).

### Assessments

The questionnaire was based on the one used by Banjo and colleagues [9]. At the beginning of the questionnaire participants had to read a short paragraph which presented an introduction of CE which summarized a longer introduction section of Banjo and colleagues because of reduced space on the paper-and-pencil questionnaire compared to the online method by Banjo and colleagues. The paragraph introduced CE drugs to be “substances which are used with the purpose to enhance one’s own cognition and that CE drugs have been developed for the treatment of cognitive decline (e.g. dementia in elderly people) or cognitive disturbance in younger subjects (e.g. attention deficit hyperactivity disorder, ADHD)”. Furthermore, primary care physicians were described as “having a key role in prescribing drugs [for CE]. Beyond that [primary care physicians] are physicians who get directly into contact with patients. Thereby, [you] are the central link between drug developments and prescribing these developed drugs to patients”.

Subsequently and identical to the original questionnaire of Banjo and colleagues, the second part of the introduction section reviewed briefly the following: “Research shows that normal decline in cognitive function in healthy individuals becomes evident in the later years of the fourth decade of life (late 30’s). Widely accepted as a normal part of aging, this cognitive decline is not a disease and moreover is distinct from the prodromic cognitive decline that precedes dementia. In a society in which one’s cognitive abilities are important determinants of self-esteem and respect from one’s peers, the normal cognitive decline of aging can be disturbing”.

Identical to Banjo and colleagues we asked for demographic data (age, gender). Beyond Banjo and colleagues, we asked for family status, hours worked per week and whether respondents were living with or without children based on the assumption that these factors could be associated with the dependent variables described below.

Furthermore, a question probing knowledge of CE, the source of knowledge about CE drugs, and the frequency being asked for a prescription of CE drugs developed by our group was asked. This was followed by a question of feeling familiar (very familiar, somewhat familiar and not familiar) with CE drugs.

A hypothetical case vignette was introduced identical to Banjo and colleagues: In this vignette, a hypothetical prescription drug approved for CE in healthy adults was introduced to the participants. The CE drug was characterized to be effective, safe and without remarkable adverse events. In order to assess the impact of patients giving reasons for requesting the drug upon physician attitudes, we presented three scenarios: a 25-year old graduate student seeking to cope with the stress of graduate school, a 45-year-old employee hoping to improve productivity, and a 65-year-old individual feeling concerns about his ability to perform everyday activities. Then, physicians were asked how comfortable they felt prescribing the hypothetical CE drug on a Likert scale (comfort level with anchors at 1 = very uncomfortable and 7 = very comfortable). Subsequently, participants were asked for the reasons that determined their comfort level.

Afterwards, we probed physicians’ attitudes towards prescribing the hypothetical CE drug and three other drugs sometimes considered to be enhancers: sildenafil, modafinil, and MPH. Using the same Likert scale we asked for their comfort level to prescribe these drugs to a 40-year-old person reporting symptoms consistent with the label indications for each drug.

The study was approved by the local Ethics Committee (Landesärztekammer Rhineland Palatine No. 837.321.08 (6318)). Participation was explained to be optional; participants gave informed consent by returning the questionnaire.

### Statistical analysis

Statistical analyses were performed using SPSS 17.0. Means are given with corresponding standard deviation (mean ± SD) and Pearson-Clopper confidence intervals (95% CI). Questions were analyzed using stepwise forward multiple logistic regression analysis with a selection level of 0.05. For evaluation of the results of the logistic regression analysis we used pseudo-R squared (Nagelkerke).

## Results

### Participants’ characteristics

Demographic data of the participating 832 physicians (= 100.0%) are given in Table 1.

**Table 1:** Participants´ characteristics

**Participants (total)**	**N = 832 (100.0%)**
Gender	
Male	567 (68.1%)
Female	259 (31.1%)
No answer	6 (0.7%)
Age	
Years (Mean ± SD)	30.0 – 81.0 years (54.3 ± 8.2)
30 – 39	40 (4.8%)
40 – 49	196 (23.5%)
50 – 59	318 (3.2%)
60 – 69	240 (28.9%)
70 – 79	11 (1.3%)
80 – 89	1 (0.1%)
Family status	
Married	734 (88.2%)
Divorced	49 (5.9%)
Single	28 (9.4%)
Widowed	12 (1.4%)
No answer	8 (1.0%)
Children	
Participants living with children	487 (58.5%)
Participants living without children	330 (39.7%)
No answer	15 (1.8%)
Certificate of Added Qualification:	
Yes	504 (60.6%)
No	328 (39.4%)
No answer	0 (0.0%)
Years working as a physician (Mean ± SD)	4.0 – 55.0 years (26.3 ± 8.5)
Years working in an own office outside of a hospital setting	
(Mean ± SD)	0.5 – 47.0 years (18.6 ± 9.3)
Living in a town (> 100.000)	114 (13.7%)
Living in a small town/village (< 100.000)	548 (65.9%)
Estimated hours of work (per week) (Mean ± SD):	8.0 – 103.0 hours (50.2 ± 14.5)

Demographic data of participants. Means are given with standard deviation (Mean ± standard deviation (SD)).

### Knowledge about CE and familiarity with this topic

#### Knowledge about CE

In total, 96.0% (n = 799 of 832) physicians had already heard about the use of substances of any kind for CE. 29.1% (n = 242 of 832) had heard about CE by friends or relatives and 33.2% (n = 276 of 832) by colleagues. 71.2% (n = 592 of 832) were informed by print media (newspapers, magazines), 33.8% (n = 281 of 832) by digital media (TV, internet), and only 4.0% (n = 33 of 832) had not yet heard about CE.

Logistic regression analysis revealed that knowledge about CE was associated with the location of the doctors’ office: Primary care physicians having their office in non-urban areas had heard about CE by colleagues significantly more often than those whose offices were in cities (p = 0.019; OR: 0.500; CI: 0.280 – 0.890; pseudo-R Squared: 0.045). Beyond that, the more years physicians worked in their office outside of the hospital setting, the greater their knowledge about CE derived from print media (p = 0.014; OR: 1.047; CI: 1.009 – 1.086; pseudo-R Squared: 0.031), and the more their general knowledge about CE (apart from the source of knowledge) (p = 0.018; OR: 0.957; CI: 0.923 – 0.993; pseudo-R Squared: 0.071). There were no associations between knowledge about CE and the following characteristics of the participants: gender, age, family status, living with children, having an additional Certificate of Added Qualification nor hours of work per week.

Physicians’ knowledge about individual substances for the purpose of CE differed substantially (see Table 2): MPH was the most widely known substance for CE, whereas caffeinated products, Ginkgo biloba as well as antidementia drugs were known only by about two thirds of the surveyed participants.

**Table 2:** Percentage of primary care physicians who know the respective substance as a drug which can be used for CE or ME

**Prescription drugs**	**%**	**N**
MPH (e.g. Ritalin®, Concerta®, etc.)	79.7	663
Ready-made AMPH tablets (e.g. Adderall®, Dexedrine®, etc.)	47.8	398
Atomoxetine (Strattera®)	23.3	193
Modafinil (Provigil®, Vigil®)	32.1	267
Antidementia drugs (e.g. Aricept®, Ebixa®/ Axura®, etc.)	61.9	515
**Illicit drugs:**		
Illicit AMPH (e.g. Speed, etc.)	49.0	408
Ecstasy	38.1	317
Cocaine	42.8	356
Other illicit psychoactive drugs	20.9	174
**OTC-drugs**		
Coffee	66.8	556
Coca Cola® (or similar)	60.5	503
Caffeinated Drinks/ Energy Drinks (e.g. Red Bull®)	64.7	539
Caffeine tablets (Coffeinum®)	61.1	508
Ginkgo biloba	59.1	492
Ephedrine	31.7	264
**Antidepressants for ME**		
Antidepressants (e.g. Prozac®, Cipralex®, Zoloft®, etc.)	42.3	352

AMPH = amphetamines; CE = cognitive enhancement; ME = mood enhancement; MPH = Methylphenidate; OTC = over the counter.

#### Being familiar with CE

Only 5.3% (n = 44 of 821) of all participants stated that they were very familiar with the topic of CE, 49.9% (n = 415 of 821) stated that they were somewhat familiar with CE and 43.5% (n = 362 of 821) stated that they were not familiar with CE.

Regarding feeling familiar with CE, there were no associations found for gender, age, years working in one’s own doctor’s office, working as a physician, hours of work per week, living with children, having an additional Certificate of Added Qualification, doctor’s office being located in a city or family status.

### Frequency of being asked by patients to prescribe a drug for CE

Regarding the estimated frequency of being asked by clients to prescribe a drug for CE purposes, 99.3% (n = 826 of 832) answered this question. 7.0% (n = 58 of 826) stated that they had been asked at least once during the last week, 19.0% (n = 157 of 826) during the last month, and 40.8% (n = 337 of 826) during the last year. 9.4% (n = 78 of 826) of the physicians had been asked more than one year ago and only 23.7% (n = 196 of 826) indicated that they had never been asked to prescribe a drug for CE by healthy subjects.

### Comfort levels of primary care physicians to prescribe drugs

a) Comfort levels regarding three scenarios

Comfort levels to prescribe CE drugs to individuals of certain characteristics (25-year old graduate student seeking to cope with the stress of graduate school, 45-year-old employee hoping to improve productivity, 65-year-old individual feeling concerns about his ability to perform everyday activities) were rated by using a 7-step Likert scale with anchors at 1 (= feeling very uncomfortable) and 7 (= feeling very comfortable).

**Figure 1:**
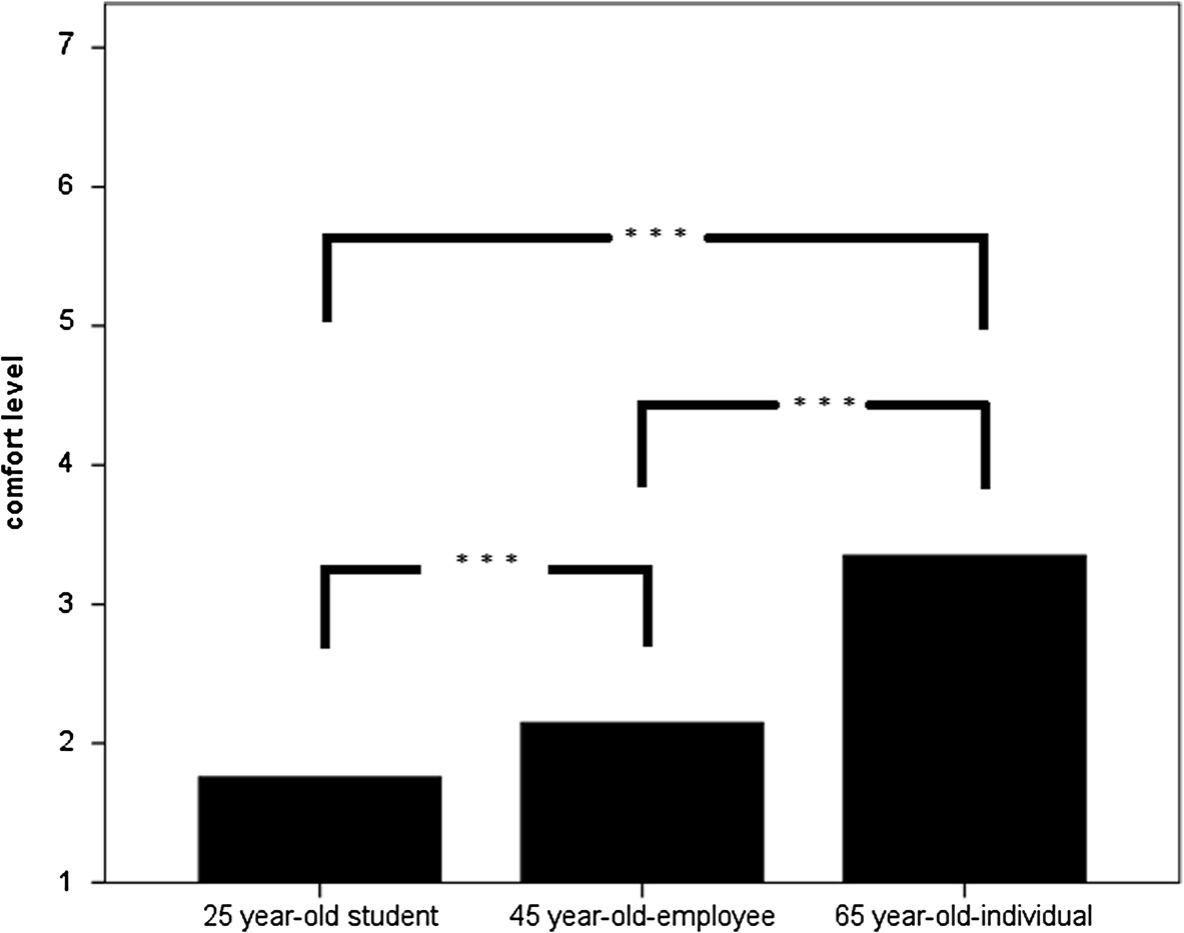
**Differences regarding comfort level to prescribe a drug to a 25-year-old graduate student, a 45-year-old-worker and a 65-year-old individual.** Means of the comfort levels were calculated using Likert scales.

Figure 1 shows differences regarding the comfort level to prescribe a drug to the 25-year-old graduate student, the 45-year-old-worker and the 65-year-old individual. Means of the comfort levels were: 1.7 ± 1.4 regarding the 25-year-old graduate student, 2.1 ± 1.5 regarding the 45-year-old-worker and 3.4 ± 2.0 regarding the 65-year-old individual. Comfort levels to prescribe were significantly higher regarding the 65-year-old-individual compared to the 25-year-old graduate student and to the 45-year-old-employee.

Logistic regression analysis revealed no significant influence regarding the tested dependent variables (age, hours of work per week, etc.) for all three scenarios.

b) Factors influencing comfort levels in prescribing CE drugs

Table 3 gives factors affecting physicians’ comfort level to prescribe a CE drug to impaired but healthy patients being 25, 45 or 65 years old. For statistical analysis see Table 3.

c) Comfort levels to prescribe a potential CE drug like modafinil or methylphenidate as compared to sildenafil

Physicians were asked how comfortable they felt prescribing sildenafil, MPH, Modafinil, or the hypothetical CE drug as described above to a 40-year-old person reporting symptoms consistent with the label indications for each drug using a 7-step Likert scale with anchors at 1 (= feeling very uncomfortable) and 7 (= feeling very comfortable).

**Table 3:** Reasons that affect attitudes for prescribing CE drugs

	**a) 25-year old graduate student seeking to cope with the stress of graduate school**	**b) 45-year-old employee hoping to improve productivity**	**c) 65-year-old individual feeling concerns about his ability to perform everyday activities**	**Statistical analysis (p-value, OR, CI)**
Availability of non-pharma-cological methods of achieving the same goals	61.5% (n = 512)	52.8% (n = 439)	31.3% (n = 260)	p < 0.001; OR: 0.071; CI: 0.039 – 0.127
Fear of misuse	60.6% (n = 504)	51.7% (n = 430)	21.8% (n = 181)	p < 0.001; OR: 0.056; CI: 0.34 – 0.91
Patient does not need the drug	54.3% (n = 452)	43.4% (n = 361)	25.1% (n = 209)	p < 0.000; OR: 0.073; CI: 0.048 – 0.112
Undermines the values of personal effort	26.9% (n = 224)	19.4% (n = 161)	8.4% (n = 70)	p < 0.001; OR: 0.027; CI: 0.15 – 0.49
To help patient succeed	13.5% (n = 114)	17.9% (n = 149)	14.8% (n = 123)	p = 0.498; OR: 0.141; CI: 0.109 – 0.181
Your cultural values	12.3% (n = 102)	11.3% (n = 94)	8.2% (n = 68)	p = 0.057; OR: 0.005; CI: 0.001 – 0.014
Fear of legal liability	10.1% (n = 84)	7.1% (n = 59)	4.4% (n = 37)	p = 0.000; OR: 0.002; CI: 0.000 – 0.014
It constitutes a form of cheating	10.1% (n = 84)	6.6% (n = 55)	2.6% (n = 22)	p < 0.001; OR: 0.006; CI: 0.002 – 0.020
To improve patients’ overall health and wellness	7.5% (n = 62)	14.4% (n = 120)	38.7% (n = 322)	p < 0.001; OR: 0.810; CI: 0.063 – 0.103
Respect for patients’ Autonomy	6.5% (n = 54)	8.7% (n = 72)	17.2% (n = 143)	p = 0.004; OR: 0.042; CI: 0.028 – 0.062
To improve daily living	6.1% (n = 51)	13.9% (n = 116)	47.6% (n = 396)	p < 0.001; OR: 0.096; CI: 0.075 – 0.122
Your religious believes	3.4% (n = 28)	3.6% (n = 30)	2.2% (n = 18)	p = 0.625; OR: 0.004; CI: 0.001 – 0.012
Patients‘ socio-economic Status	3.0% (n = 25)	6.6% (n = 55)	4.8% (n = 40)	p = 0.044; OR: 0.091; CI: 0.057 – 0.193
Drug is age-appropriate	1.6% (n = 13)	4.2% (n = 35)	20.7% (n = 127)	p < 0.001; OR:0.040; CI: 0.024 – 0.065

Reasons affecting attitudes for prescribing CE drugs of the surveyed primary care physicians. Statistical analysis was carried using p-values, odds ratios (OR) and confidence intervals (CI).

**Figure 2:**
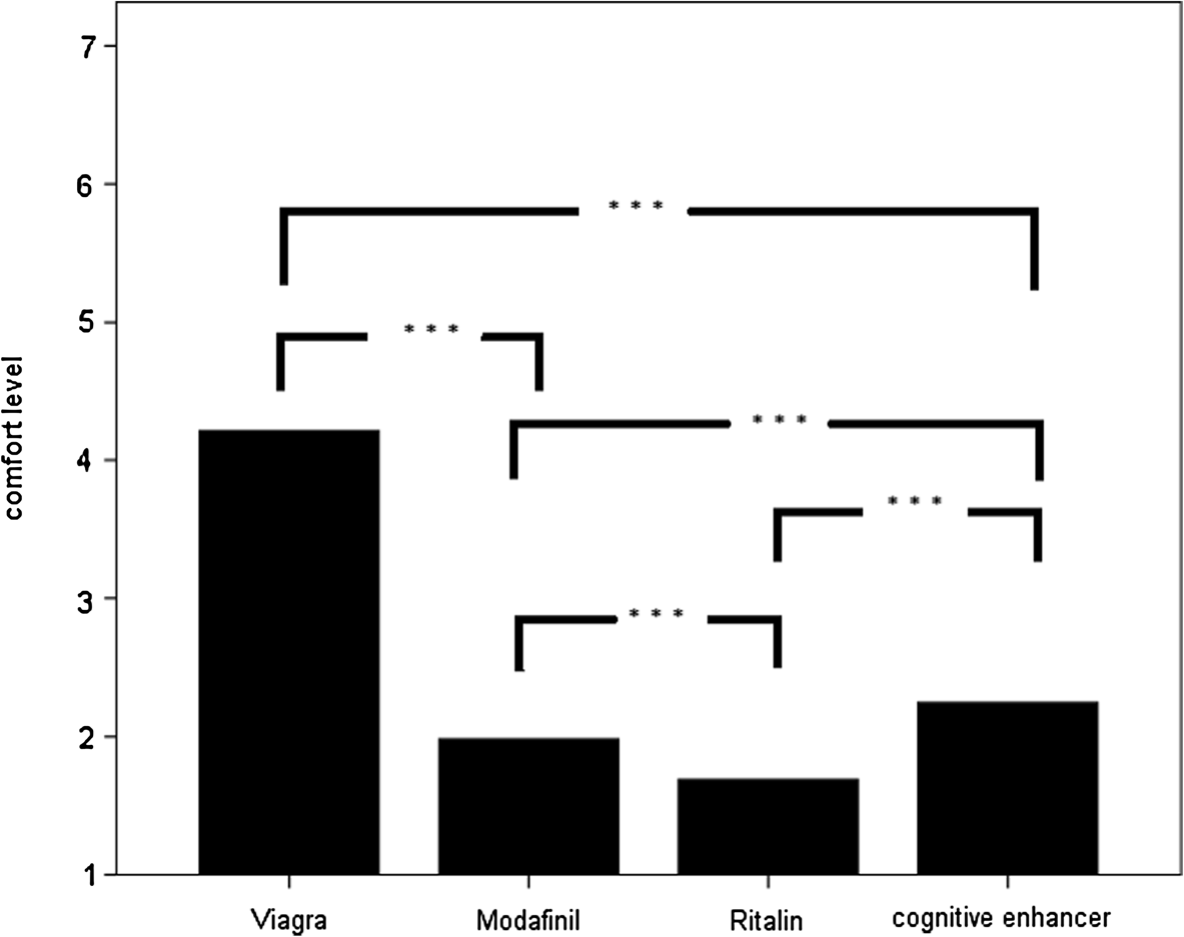
Comfort levels to prescribe Viagra®, Modafinil, Ritalin®, Cognitive enhancer using Likert scales (comfort level with anchors at 1 = very uncomfortable and 7 = very comfortable).

Figure 2 shows comfort levels of 4.2 ± 2.0 regarding the comfort level to prescribe Viagra®, 2.0 ± 1.5 to prescribe Modafinil, 1.7 ± 1.3 to prescribe Ritalin® and 2.3 ± 1.6 to prescribe a potential CE drug.

There were significant differences between the disposition of the physicians to prescribe the different types of drugs. Physicians felt significantly more comfortable in prescribing Viagra® then prescribing modafinil, Ritalin® or the hypothetical CE drug. In addition physicians felt much more comfortable in prescribing the hypothetical enhancer than choosing modafinil or Ritalin®.

With respect to comfort levels of physicians regarding the prescription of sildenafil, modafinil, MPH or a hypothetical CE drug, logistic regression analysis revealed that male primary care physicians felt significantly more comfortable prescribing sildenafil than female physicians (p = 0.003; OR: 0.463; CI: 0.159 – 0.767; pseudo-R Squared: 0.026) as well as primary care physicians having their doctor’s office in a city showed a higher comfort level for prescribing sildenafil (p = 0.048; OR: 0.321; CI: 0.003 – 0.640; pseudo-R Squared: 0.026). Regarding comfort levels of prescribing sildenafil, there were no associations with age, years working as a physician, years working in one’s own doctor’s office, hours of work per week, family status, living with children, and the location of one’s own doctor’s office.

Regarding modafinil, male primary care physicians and physicians having their office in a city felt more comfortable prescribing modafinil (p = 0.001; OR: 0.573; CI: 0.227 – 0.918 and p = 0.010; OR: 0.484; CI: 0.118 – 0.850; pseudo-R Squared: 0.045 respectively). There were no associations with age, years worked as a physician, years worked in own doctor’s office, hours worked per week, family status, living with children, location of doctor’s office.

For MPH and the hypothetical CE drug, we found the same positive association with male physicians as with sildenafil and modafinil (MPH: p = 0.030; CI: 0.040 – 0.786; OR: 0.413, pseudo-R Squared: 0.029; hypothetical CE drug: p = 0.034; CI: 0.028 – 0.682; OR: 0.354, pseudo-R Squared: 0.035). Further associations were not found for MPH as well as the putative CE drug (MPH: age, years worked as a physician, years working in one’s own doctor’s office, hours worked per week, family status, living with children, additional Certificate of Added Qualification, location of doctor’s office. Putative CE drug: age, years worked as a physician, hours worked per week, family status, living with children, additional Certificate of Added Qualification, location of doctor’s office).

## Discussion

This study investigated knowledge of, attitudes towards, familiarity with, frequency of being asked for prescriptions for, and comfort levels with prescribing CE drugs among 832 primary care physicians in Germany. The study showed an especially high knowledge level regarding CE among the participating subjects with MPH being the most widely known substance for CE. In contrast to this high knowledge level, only 5.3% of the physicians stated that they were very familiar with CE, and 43.5% described themselves as being not familiar with the subject. 40.8% of the surveyed primary care physicians had been asked for a prescription for CE during the last year followed by 19.0% which had been asked during the last month and 7.0% during the last week. Comfort levels to prescribe CE drugs are low among the surveyed physicians and significantly lower than to prescribe sildenafil (Viagra®). Another main finding is that comfort levels to prescribe a CE drug are mainly affected by the age of the asking subject followed by the availability of non-pharmacological alternatives, fear of misuse of the prescribed drug and the missing necessity of needing the drug.

We demonstrated that nearly all surveyed primary care physicians (96.0%) reported that they knew about the possibility of pharmacological CE. This is significantly higher proportion than among students in previous studies. Two studies among nearly 10,000 students reveal that about 80% of surveyed students knew about this possibility of CE [12,13,19]. Furthermore, 81.4% of German students stated to know about using substances of any kind for CE which was significantly higher in university than in high school students [13]. We can only speculate about the reasons; one reason may be the higher age of the surveyed primary care physicians going along with an increase of professional expertise being probably associated with age. In distinction from our previous study among students, in this study we were not able to detect differences regarding sex [13]. Regarding stimulants, 39.8% of the students knew about prescription stimulants for CE and 57.9% about illicit stimulants for CE [12]. Primary care physicians were less informed about the use of illicit stimulants but more informed regarding prescription stimulants. Even if this and previous studies examine the knowledge of CE, there are no comparable data regarding the single substances for CE.

Although nearly all physicians had heard about the possibility of CE, only about half of the physicians felt they were not familiar with the topic of CE, and only a minority of physicians felt very familiar with CE. As compared to Banjo and colleagues, we obtained comparable results for feeling very familiar with CE (Banjo and colleagues: 4.0%, present study: 5.7%) [9]. However, Banjo and colleagues reported a higher percentage of physicians (57.0% vs. 43.5% in our study) feeling not familiar with CE and a respective lower percentage (39.0% vs. 49.9% in our study) feeling somewhat familiar with CE. We can only speculate about possible causes for these differences. One might be the different time point of assessment (2009 in the study by Banjo et al. and 2011 in our study) or differences in information systems between Canada and Germany. Another explanation for the low level of familiarity may be the fact that prescription drugs for somatic disorders are much more prevalently prescribed than prescription drugs for CE belonging to the group of drugs prescribed for mental disorders by primary care physicians [1].

Compared to Hotze and colleagues, the percentage of primary care physicians who reported being asked for a prescription of a drug for CE during the last week was considerably lower in our study: 62% in the survey by Hotze and colleagues receive requests “to prescribe interventions for what they view as enhancement purposes” monthly and 12.0% daily as compared to 7.0% during the last week, 19.0% during the last month, and 41.0% during the last year in our study presented here. We can only speculate about the reasons for his difference. Age and sex of the participants of the two studies is comparable (Mean age in our study: 54.3 years, Hotze et al.: 52.6 years; sex in our study: male: 68.1%, female: 31.1%, Hotze et al.: male: 72.0%, female: n.a.). Unfortunately, neither Hotze and colleagues nor our study describes the requesting individuals (age, sex, students, workers, etc.). One possibility is that the requesting individuals in the study of Hotze and colleagues had different characteristics than the requesting individuals remembered by the surveyed primary care physicians in our study. Furthermore, in the introduction section of our questionnaire we defined CE drugs to be “substances which are used with the purpose to enhance one’s own cognition and that CE drugs have been developed for the treatment of cognitive decline (e.g. dementia in elderly people) or cognitive disturbance in younger subjects (e.g. attention deficit hyperactivity disorder, ADHD)”. Hotze and colleagues asked about “how often patients requested medicine or services that the physicians considered to be for enhancement rather than therapy” [2]. Thus, the definition in our study is much more precise and tight than in the study of Hotze and colleagues and may be the main reason for the significantly higher requesting rates in Hotze and colleagues.

Using vignettes of patients requesting a physicians’ prescription of a CE drug, we probed participants about their behaviour in case of a healthy 25-, 45- and 65-year old individual having cognitive problems and therefore participants having a reason for prescribing a CE drug. We found age of the requesting patient/client to be the main factor determining comfort level of the surveyed primary care physicians. However, even if the aim of all three scenarios is cognition enhancement, the reasons for the requests are different (to cope with stress at graduate school, improve productivity at work, leading an active life to counteract subjective cognitive decline). This has to be considered when interpreting that age of the requesting person is the decisive associated factor. Further participants’ characteristics were found to play no role (sex, etc.). This is in line with previous results of Banjo and colleagues who found that the age of the requesting patient/client as being a key determining factor [9]. Furthermore, they found the same in case of a healthy 25-, 45- and 65-year old individual without any cognitive problems and with reasons for requesting a CE drug [9].

We found that fear of misuse, availability of non-pharmacological methods of achieving the same goals and the fact that the requesting individual does not need the drug to be the most relevant reasons affecting physicians comfort levels of prescribing CE drugs in case of the 25-year old college student and the 45-year old employee. These results confirm the results of Banjo and colleagues [9]. Beyond that, in our study as well as in Banjo and colleagues the factors to improve patient’s overall health and wellness, to improve daily living and the assumption/fact that the drug is age-appropriate were the most crucial factors regarding the prescription of a CE drug to the 65-year old individual feeling concerns about his ability to perform everyday activities. Fear of legal liability as well as the aspect that the use of CE drugs constitute a form of cheating played a very minor role in both studies. This is in line with previous results of our group: In an interview study about reasons of students justifying their use of stimulants for CE compared to caffeine we found that legal aspects play a very minor role for them [22]. Interestingly, for student users as well as potential prescribers (primary care physicians) legal aspects play a minor role.

The last set of questions was about a comparison of sildenafil, MPH, modafinil and a hypothetical CE drug prescribing to patients having the label indication. We found that the highest comfort level for prescribing these agents was for sildenafil (comfort level 7 = 13.1%) compared to MPH (comfort level 7 = 1.8%), modafinil (comfort level 7 = 2.5%) and the hypothetical CE drug (comfort level 7 = 1.9%). These results are similar to those of Banjo and colleagues [9] and show that there is some degree of similarity in transnational attitudes. Furthermore, when asked to freely respond on their answers, Banjo and colleagues show that the surveyed physicians stated to be more familiar with sildenafil and that the latter should have a better safety profile. Further comments show the primary care physicians being afraid about the abuse potential of stimulant drugs.

Beyond that, we found that male primary care physicians had a higher comfort level to prescribe sildenafil, modafinil, MPH or a hypothetical CE drug to patients having an indication for a prescription. This is in line with previous results by Ponnet and colleagues searching for determinants of physicians to prescribe MPH for CE using the theory of planned behaviour (TPB). They found that gender influenced attitudes towards prescribing MPH for CE, too: Compared to male physicians, female physicians had more negative attitudes towards prescribing MPH for CE [23]. However, they used a vignette presenting a healthy university student and did not probe for older patients/clients with or without reasons/symptoms for a prescription.

Finally, primary care physicians have a crucial role of the supply of prescription drugs for CE, they are meant to be gatekeepers to the medical system and they are the first who are contacted by the general public searching for a physicians’ prescription. However, at the present time the prescription of CE drugs by primary care physicians is much less prevalent than the prescription of somatic medication to patients [1].

At present primary care physicians have to decide what to do on their own regarding CE. Although nearly all of them reported that they knew about the phenomenon of pharmacological CE there is a lack of guidelines aimed at primary care physicians. One possibility is that the existing guidelines to neurologists for adult and paediatric populations could be adopted. The “Guidance of the Ethics, Law and Humanities Committee” for neurologists provides neurologists with an overview of ethical, legal, and social issues surrounding CE as well as practical guidance for responding to an adult patient’s request for CE drugs developed by Larriviere and colleagues [24]. These guidelines propose that neurologists have no obligation to prescribe CE drugs and may ethically refuse a prescription. They should exercise “their clinical and ethical judgment to decide whether to prescribe medications for neuroenhancement”. It would be “ethically permissible for neurologists to prescribe such therapies, provided that they adhere to well-known bioethical principles of respect for autonomy, beneficence, and nonmaleficence” [24].

Beyond that, “Ethical, legal, social, and neurodevelopmental implications” have been developed for pediatric CE [25]. Graf and colleagues stated that prescribing CE drugs to children and adolescents without a neurological diagnosis is not justifiable. In “nearly autonomous adolescents” this dogma should be weaker, but prescribing CE drugs should be not advisable “because of numerous social, developmental, and professional integrity issues” [25].

These position papers are primarily directed at neurologists, but the conclusions are indeed relevant to primary care physicians as well. What is missing from the discussion is the development of a more general set of guidelines that can apply to all physicians – neurologists, primary care physicians, and others – that will assist them in their decision-making with respect to prescribing CE. Much more data about the phenomenon of CE is needed. At least, medical education and post-graduate education of physicians should contain information about the pro-cognitive limitations, the fact of pro-cognitive placebo-effects and the relevant side effects as well as the safety profile of potential CE drugs as well as ethical and social implications. Clients claiming for a CE prescription should be elucidated by their physicians.

A few questions for further studies arise based upon the presented data and should be addressed in further studies to inform the debate about CE: We do not know if the clients asking for a prescription are younger students needing help to perform better in school and university or older ones complaining cognitive decline. This would be an important additional piece of information that could be used to characterize claims for CE and the prescription of CE drugs. Therefore, further studies among primary care physicians should address the question of characteristics of patients asking for a CE prescription. Furthermore, the present study does not assess if the clients ask for a special drug (e.g. MPH) which they want to get prescribed or if they ask for general pharmaceutical cognitive help. In this respect we do not know what requested primary care physicians do after having been requested for a prescription. The behaviour after being asked for a prescription should be addressed in further studies as well. Beyond that, we did not ask for interventions primary care physicians do after being asked for a prescription e.g. counselling regarding alternative possibilities to enhance cognition or mood and if they explain the small pro-cognitive effect as well as the (dangerous) side effect profile of the present drugs. To address these questions and questions to the close context in case of being asked for prescribing a CE drug may be contents for qualitative research (interview studies) among primary care physicians. Unfortunately, these questions cannot be addressed by the use of anonymous questionnaires (neither paper-and-pencil, nor web-based). Therefore, in depth interviews of primary care physicians should be done.

Some limitations of the study are worth identifying. A general problem of anonymous surveys is the possibility of misunderstanding questions and the interpretation of the questions by the participants. Together with the use of case vignettes this may lead to a certain kind of fuzziness of data obtained. Furthermore, the relevance perceived by the participants may influence the answers obtained. The more important aspect regarding understanding and interpretation of this survey may be the socially undesirable behaviour of misusing substances to enhance cognition which can be regarded to be comparable to the use of drugs for physical enhancement. Answering questions regarding such a stigmatizing subject – even if the survey is anonymous – may lead to socially desired answers depicting a bias of the present data.

The sample of primary care physicians of Rhineland-Palatine is large, but is neither representative of Germany nor other countries. Furthermore, the response rate was 30.2%. This response rate of only 1/3 means a selection bias. We can only speculate about the reasons of non responding to the questionnaire (e.g. lack of time, feeling that the topic is not important, socially non-desired opinions, etc.). These aspects make it difficult to generalize from the results.

Beyond that, the logistic regression analysis was the most appropriate method to analyse the data of this survey study. However, several times ORs are quite close to 1.0 and the analysis of pseudo-R Squared are smaller than 0.1 which limits the explanatory power of the analysis.

## Conclusion

The data presented in this study confirm and extend previous studies of physician attitudes towards prescribing enhancement pharmaceuticals to physicians in Germany. Given the different social, legal and medical contexts in Germany, the present results are important in demonstrating that the general trends of physician attitudes towards enhancement are relatively stable: physicians view themselves as gatekeepers and are generally less comfortable prescribing CE for younger populations than for older individuals. Despite these observations, the study highlights the need for further education of physicians about the biological, ethical, and social consequences of CE use, and suggests that an organized international effort for outreach to physicians is both appropriate and timely.

## Abbreviations

AMA: American Medical Association; AMPH: (Prescription) Amphetamine(s); AQ: Anonymous questionnaire; ADHD: Attention deficit/hyperactivity disorder; CE: (Pharmacological) cognitive enhancement; CI: Confidence interval; ME: (Pharmacological) mood enhancement; MPH: Methylphenidate; OR: Odds ratio; OTC: Over the counter; RCT: Randomized controlled trial; RRT: Randomized response technique; SD: Standard deviation; TPB: Theory of planned behaviour.

## Competing interests

All authors declare to have no competing interests.

## Authors’ contributions

AGF, KL and PBR participated in the conception and design of the study. AGF, CP and ES monitored data collection. AGF, KL, CP analysed and checked the data calculation. All authors participated in data interpretation, drafting, and revising the manuscript. All authors read and approved the final manuscript.

## Authors’ information

AGF, ES, CP and KL belong to the Department of Psychiatry and Psychotherapy, University Medical Centre Mainz, Germany. KL is a Professor for Psychiatry and Psychotherapy and the the head of the Department of Psychiatry and Psychotherapy. AGF is a research fellow at the Department for Psychiatry and Psychotherapy and has recently received a Professorship for Medicine in Social Work and Education at the University of Neubrandenburg; ES and CP are doctoral students of the Department of Psychiatry and Psychotherapy. PBR is a Professor for Neuroethics at the University of British Columbia (UBC), he is trained in neurobiology of behavioral states and the molecular underpinnings of neurodegenerative disease. He focuses his research in the area of neuroethics at the National Core for Neuroethics at the UBC in Vancouver (Canada).

## Pre-publication history

The pre-publication history for this paper can be accessed here:

http://www.biomedcentral.com/1471-2296/15/3/prepub
